# Guillain–Barre syndrome with pleocytosis requires workup for cerebral disease

**DOI:** 10.1002/ccr3.7321

**Published:** 2023-05-04

**Authors:** Josef Finsterer, Fulvio A. Scorza

**Affiliations:** ^1^ Neurology and Neurophysiology Center Vienna Austria; ^2^ Disciplina de Neurociência Universidade Federal de São Paulo/Escola Paulista de Medicina (UNIFESP/EPM) São Paulo Brazil

## ETHICS STATEMENT

This article is based on previously conducted studies and does not contain any new studies with human participants or animals performed by any of the authors.

## CONSENT

Written informed consent was obtained from the patient for publication of the details of their medical case and any accompanying images.

Letter to the editor

With interest we read the article by Tran et al. on a 60‐year‐old‐male who developed progressive, ascending muscle weakness and sensory disturbances of the upper and limbs 3 days after having been tested positive for SARS‐CoV‐2.^1^ COVID‐19 took a mild course and manifested only with nausea, fever, and myalgias.^1^ The patient was diagnosed with Guillain–Barre syndrome (GBS), subtype acute, motor and sensory, axonal neuropathy (AMSAN) an was treated with steroids and plasmaphereseis (PLEX).^1^ Despite these measures, the patient deteriorated and developed quadriplegia, respiratory insufficiency, respiratory distress syndrome (ARDS) requiring intubation and mechanical ventilation, and septic shock.^1^ The patient was lastly transferred to a long‐term acute care hospital for continued management and ventilator weaning.^1^ The study is compelling, but has limitations that are cause of concerns and should be discussed.

We disagree with the notion that SARS‐CoV‐2 related GBS is a rare disease.^1^ On the contrary, there is increasing evidence that GBS can be a complication in adult and pediatric SARS‐CoV‐2 infections. Several hundred cases of SARS‐CoV‐2 related GBS have been reported worldwide since onset of the pandemic.

Regarding the cerebrospinal fluid (CSF) findings, we should know why the patient had pleocytosis of 27/3 cells.^1^ We should know whether all infectious causes of pleocytosis, including bacterial, viral, fungal, and parasitic meningitis and encephalitis were adequately ruled out. In patients with GBS the CSF cell count is usually normal (“dissociation cyto‐albuminuqe”). We should know the results of follow‐up CSF examination.

A limitation of the study is that the CSF was not investigated for cytokines, chemokines, glial factors, and 14‐3‐3. These parameters have been reported elevated in patients with neuro‐COVID, particularly in patients with SARS‐CoV‐2 related GBS.^2^

Another limitation of the study is that the patient did not undergo cerebral MRI with contrast medium. Cerebral MRI was indicated as there was mild pleocytosis and as GBS can manifest with Bickerstaff brainstem encephalitis. It is also feasible that the patient had autoimmune encephalitis (AIE) in addition to AMSAN. Were AIE antibodies positive in the CSF?

It is unclear why the patient received steroids in addition to PLEX. From previous studies it is known that adding steroids to PLEX does not result in an additional benefit regarding the outcome. It is also unclear why the patient did not receive intravenous immunoglobulins (IVIGs). It is also unclear when PLEX was started. Starting treatment for GBS early is crucial as it can significantly improve the outcome of these patients.

We disagree with the notion that the patient had undergone “electromyography” (EMG).^1^ The patient had undergone nerve conduction studies (NCS), which is a neurophysiological technique different from that of needle EMG.

Overall, the interesting study has limitations that call the results and their interpretation into question. Addressing these issues would strengthen the conclusions and could improve the status of the study. In patients with SARS‐CoV‐2‐related GBS and pleocytosis it is critical to rule out infectious or immunological central nervous system disease.

## AUTHOR CONTRIBUTIONS

**Fulvio A Scorza:** Investigation; methodology; resources.

## FUNDING INFORMATION

No funding was received.

## CONFLICT OF INTEREST STATEMENT

The author declares that the research was conducted in the absence of any commercial or financial relationships that could be construed as a potential conflict of interest.

## Data Availability

Data that support the findings of the study are available from the corresponding author.

## References

[1] Tran DH , Basra D , Bilgrami Z , et al. Guillain‐Barre syndrome secondary to COVID‐19 infection: a case report. Clin Case Rep. 2023;11(4):e7104. doi:10.1002/ccr3.7104 37006840 PMC10062311

[2] Gigli GL , Vogrig A , Nilo A , et al. HLA and immunological features of SARS‐CoV‐2‐induced Guillain‐Barré syndrome. Neurol Sci. 2020;41(12):3391‐3394. doi:10.1007/s10072-020-04787-7 33006723 PMC7530349

